# Predicting the Activity Level of the Great Gerbil ( *Rhombomys opimus* ) via Machine Learning

**DOI:** 10.1002/ece3.71452

**Published:** 2025-05-26

**Authors:** Fan Jiang, Peng Peng, Zhenting Xu, Yu Xu, Ding Yang, Shouquan Chai, Shuai Yuan, Limin Hua, Dawei Wang, Xuanye Wen

**Affiliations:** ^1^ Center for Biological Disaster Prevention and Control National Forestry and Grassland Administration Shenyang China; ^2^ College of Grassland, Resources and the Environment Inner Mongolia Agricultural University Hohhot China; ^3^ Key Laboratory of Grassland Ecosystem of the Ministry of Education College of Grassland Science, Gansu Agricultural University Lanzhou China; ^4^ Western Agricultural Research Center Chinese Academy of Agricultural Sciences Changji China; ^5^ State Key Laboratory for Biology of Plant Diseases and Insect Pests Institute of Plant Protection, Chinese Academy of Agricultural Sciences Beijing China

**Keywords:** extreme learning machine, machine learning, neural networks, population monitoring, *Rhombomys opimus*

## Abstract

The great gerbil ( *Rhombomys opimus* ) is a pest rodent that is widely distributed in Eurasia, and assessing its outbreak risk and instituting timely population control are very important for protecting vegetation and human health. Because traditional assessment methods are difficult to monitor and cannot effectively predict the population growth trend of *R. opimus* , an *R. opimus* activity prediction model was constructed using the particle swarm optimization algorithm‐extreme learning machine (PSO‐ELM). First, data for 13 factors influencing *R. opimus* growth, such as those related to the environment, vegetation, and activity in the previous year, at 46 *R. opimus* monitoring sites in China from 2020 to 2022 were selected. Second, principal component analysis was used to reduce the dimensionality of the 92 sets of collected data to six principal components, thus eliminating the correlation between the indicators. Third, after dimensionality reduction, the data were divided into a training set (80 sets of data) and a test set (12 sets of data) for model training and simulation, and the prediction results of the PSO‐ELM model and back propagation model were compared. The simulation results revealed that the PSO‐ELM model has a stronger convergence ability and higher prediction accuracy for the activity level of *R. opimus* in fall (91.67%). In this study, a new method is provided for surveying pest rodents. The proposed method provides an auxiliary means of managing *R. opimus* . We will continue to improve the sample data in future work to obtain more accurate predictions.

## Graphical abstract

The particle swarm optimization algorithm–extreme learning machine was utilized to develop the hazard model for the great gerbil.

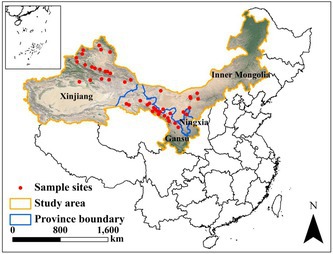

## 1 Introduction

In the past few decades, how to manage pest rodents has been a main challenge facing the development of grassland resource protection and desert afforestation in China (Zhang et al. 2020). A key difficulty is that we cannot predict the numbers of sudden outbreaks of pest rodents (Wan et al. 2022; Nance et al. 2023). Historically, the struggle between humans and mice for food and living space has been ongoing for thousands of years, and many rodent control agents and methods for trapping mice have been developed (Quy et al. 1995; Watt et al. 2005; Hacker et al. 2016; Wen, Yuan, et al. 2022; Awoniyi et al. 2024). Regrettably, we are still unable to implement effective measures to prevent the large‐scale emergence of harmful rodents, which would help minimize the economic losses they inflict on the forestry and grassland industries.

The great gerbil ( *Rhombomys opimus* ), a pest rodent distributed in Central Asia, currently has the largest distribution area of all pest rodents in China (Xu et al. 2015). The detrimental impacts of *R. opimus* are predominantly manifested in two critical dimensions. On the one hand, *R. opimus* consumes desert vegetation to such an extent that the vegetation loses water and dies, while its burrowing behaviors significantly decrease surface plant coverage and aggravate regional desertification (Dang et al. 2022). On the other hand, *R. opimus* is the main host of rat plague, leishmaniasis (*Leishmania major*), and the Karimabad virus (Shiravand et al. 2019; Li et al. 2022), which pose notable threats to human health. At present, *R. opimus* is mainly surveyed manually by setting up mouse traps or investigating infested plants; these methods are not only inefficient but also pose a high risk of plague infection.

To improve survey methods, infrared cameras can be used for monitoring *R. opimus* (Wen et al. 2020). However, due to the high cost of use and the need for subsequent maintenance, infrared cameras are not ideal for monitoring surveys of large areas. Therefore, a new method that can reduce survey costs while providing a relatively high survey accuracy rate for the largest possible monitoring area is urgently needed.

The rapid development of computer technology in recent years has enabled the reproductive patterns of rodents to be discovered through machine learning methods; these patterns can be used to predict rodent occurrence. Langton (1989) proposed that a neural network can be used to predict the population size of pest rodents. However, since then, neural networks have typically been used to analyze the social behavior of rodents (Isik and Unal 2023). Lu et al. (2017) used a back propagation (BP) neural network to predict the population sizes of three species of desert pest rodents and obtained very good prediction results, but the number of samples used in the study was small, and the proposed method was suitable only for application in some desert areas. In addition, the BP neural network has many parameters, requires large amounts of data, many calculations, and a long calculation time, and is hindered by gradient disappearance and gradient explosion (Abuqaddom et al. 2021; Al‐Abri et al. 2021); thus, this method has not yet been widely used. Therefore, according to these studies, a model that is suitable for more regions and has accurate predictions and greater stability is needed.

Particle swarm optimization (PSO) is a binary particle swarm algorithm. Compared with other intelligent algorithms, PSO differs by simulating a society (Bansal 2019). We can think of the optimization problem as the process of a flock of birds looking for something to eat, for which each bird is a hidden solution to the problem, and the thing birds are looking for is the optimal solution to the problem. Once the birds find what they are looking for, the optimal solution of the problem is found. The PSO algorithm has the characteristics of fast convergence velocity, simple structure, convenient adjustment, easy program implementation, and strong memory (Jiang et al. 2004; Ang et al. 2020); therefore, the PSO algorithm is applied in the optimization process of neural networks. An extreme learning machine (ELM) is a single hidden layer feedforward neural network (Mujal et al. 2021). It consists of three main parts: an input layer, a hidden layer, and an output layer. Unlike BP neural networks, ELMs do not require the backpropagation of errors. They are characterized by fewer adjustable network parameters, thus requiring less computational effort for model training, enabling faster training times, and reducing the likelihood of model overfitting. At present, the PSO‐ELM model has achieved satisfactory results in predicting fruit yields (Lu et al. 2022) and providing early warnings for geological disasters (Yuan et al. 2023).

In summary, on the basis of many surveys regarding the distribution and activity of *R. opimus* in China, the PSO‐ELM can be utilized to develop a predictive model for activity levels associated with *R. opimus* . One objective of this study is to mitigate the reliance on field traps and the manual counting of infested plants for assessing *R. opimus* population sizes, thereby reducing the risk of surveyors contracting rodent‐borne diseases in areas where *R. opimus* is prevalent. Another objective was to optimize monitoring methods for *R. opimus* so that surveyors can effectively predict activity levels during critical seasons of rodent activity and sudden outbreaks of nuisance rodents can be controlled.

## 2 Materials and Methods

### 2.1 Study Area

The study sites cover four provinces in northwestern China, that is, the Inner Mongolia Autonomous Region, Gansu Province, Ningxia Hui Autonomous Region, and Xinjiang Uygur Autonomous Region (36°58′ N to 47°71′ N and 82°56′ E to 108°66′ E). Monitoring records for the past several years have confirmed that these areas are the main active areas of *R. opimus* in China (Wen, Zhao, et al. 2022). The study area includes a variety of suitable habitats (e.g., steppe, Gobi and desert) for *R. opimus* . These habitats mainly have temperate desert and steppe continental climates, with scarce rain, very dry conditions, strong solar radiation, high temperatures, and vigorous evaporation. The temperature varies rapidly; the temperature difference between day and night ranges from 20°C to 30°C (Indoitu et al. 2012). In addition to *R. opimus* , other common rodents include *Meriones meridianus* , *Dipus sagitta* , *Eolagurus luteus* , and *Lepus tolai* (Chen et al. 2017).

**FIGURE 1:**
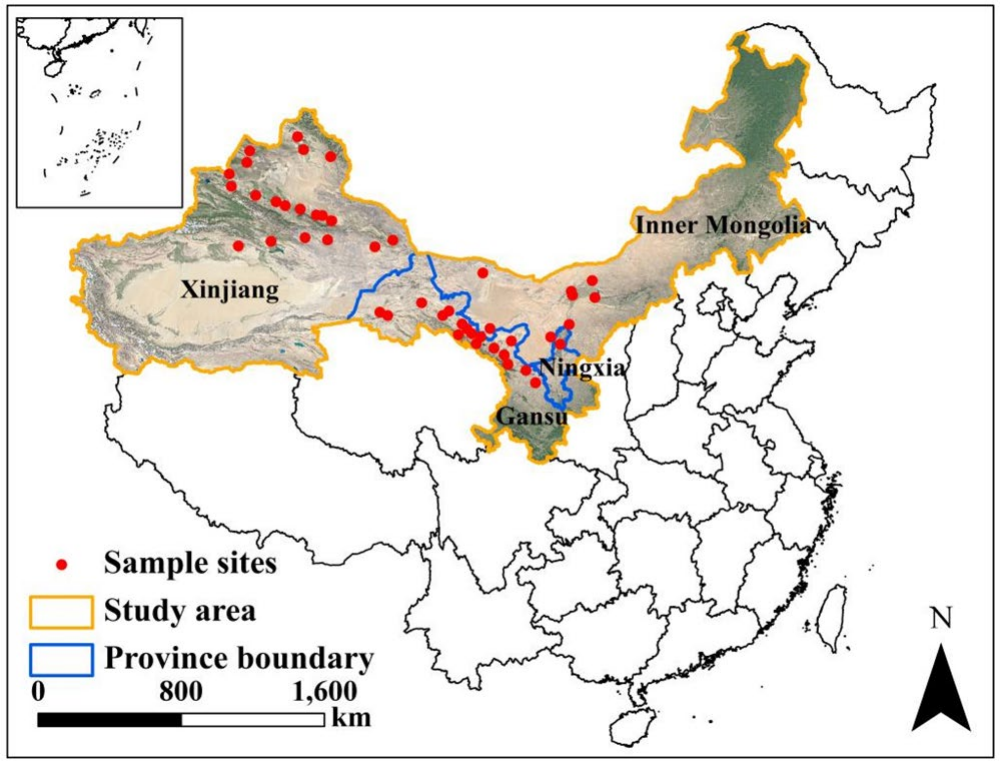
The monitoring sites of *R. opimus* in northwestern China (distributed in the four provincial‐level administrative regions of Inner Mongolia, Gansu, Ningxia, and Xinjiang). The yellow line represents the range of the study area, and the blue line represents the boundary of each provincial‐level administrative region.

Within the study area, we selected 46 1‐ha sample plots to conduct surveys of the activity levels of *R. opimus* (Figure 1). All plots were confirmed to have recorded occurrences of *R. opimus* in 2019, and each plot was spaced at least 100 km^2^ apart, positioned as far away as possible from human settlements. This arrangement was intended to effectively represent the vegetation and environmental conditions of various *R. opimus* occurrence areas in China. During the trial period, no artificial disturbances, such as hand trapping or the release of poisonous baits, occurred in these 46 sample plots during the fall (September–November in the Northern Hemisphere) each year from 2020 to 2022. Rodent traps were constructed from medium‐sized board clips (8 × 15 cm), with every 25 traps arranged in a straight line, spaced 5 m apart, and with a row spacing of 20 m, resulting in a total of 100 traps arranged in 4 rows side by side. The traps were collected after 24 h of placement, and only the number of captured *R. opimus* individuals was recorded, without counting other rodent species (Liu et al. 2018); any loss of traps was considered an uncaptured rodent, and traps experiencing > 10 losses were systematically relocated to repeat the survey. Additionally, 100 shrubs were randomly sampled within the sample plots, and their branches were examined to record the number of shrubs that were fed on by *R. opimus* .

### 2.2 Model Structure and Algorithm Process

The prediction of *R. opimus* activity via the PSO‐ELM model involves the following two steps: (1) various indices that affect the activity level of *R. opimus* were analyzed and selected and (2) a prediction model for the activity level of *R. opimus* was constructed, trained, and predictions were generated.

**FIGURE 2:**
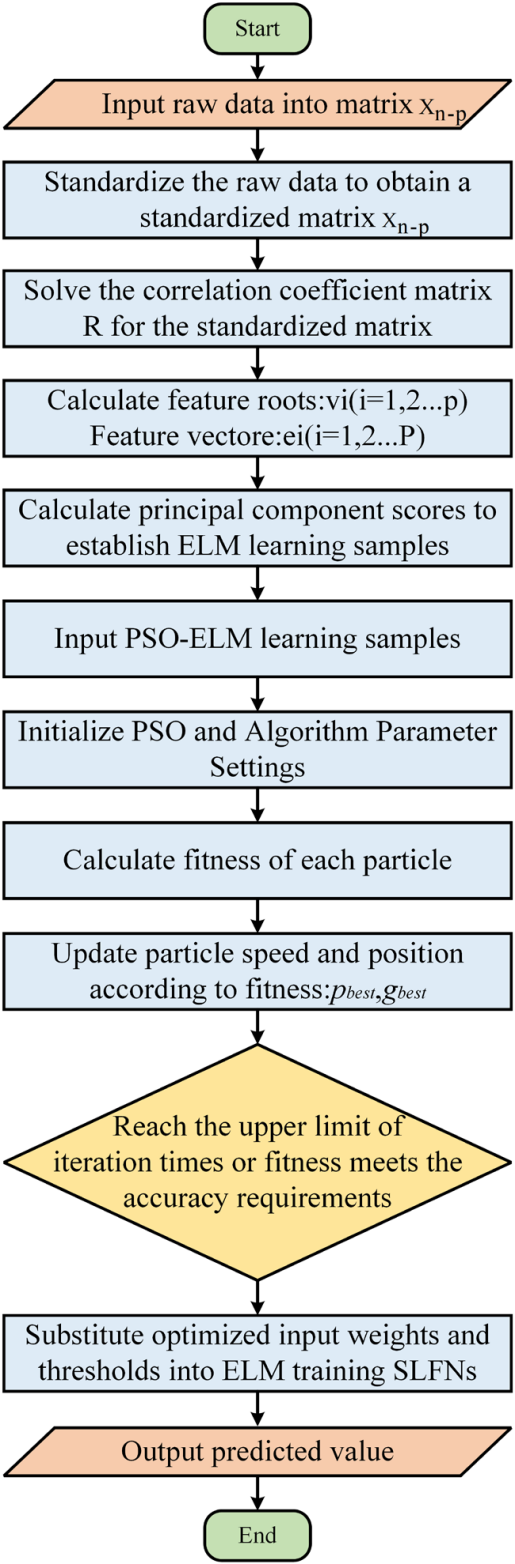
Principal component analysis, particle swarm optimization, and extreme learning machine flowchart.

The technical process determined by the above steps is shown in Figure 2. First, each input influencing factor is standardized. Second, dimensionality reduction analysis is performed according to principal component analysis (PCA) and the procedure to determine the eigenvectors and eigenvalues of the covariance. Third, the contribution rates are arranged in descending order, and the eigenvectors are rearranged. Fourth, the principal components with cumulative contribution rates greater than 0.85 are screened out, and the samples are re‐constructed for assembly modeling. The data are divided into the training set and the test set. The training set is used to train the optimized assembly model. After the training is complete, a new sample set is input to obtain the final prediction result. All the above procedures were programmed in MATLAB 2021b.

### 2.3 Selection of Modeling Factors

Based on previous studies in biology and ecology, a total of 13 modeling factors in three categories, namely environment, vegetation, and previous year activity, which are closely related to the life and reproduction of *R. opimus* , were included (Neronov and Lushchekina 1997). The selection of environmental data was mainly based on our early niche study on *R. opimus* (Wen, Zhao, et al. 2022), including: the topsoil gravel content, elevation, topsoil silt fraction, topsoil organic carbon, topsoil pH (H_2_O), annual mean temperature, precipitation of the wettest month, annual precipitation, mean temperature of the wettest quarter, precipitation of the warmest quarter, and mean temperature of the driest quarter. The vegetation data were obtained from field surveys at 46 monitoring sites. The age of the vegetation in the living area of *R. opimus* was summarized; that is, shrubs less than 5 years old were considered young, shrubs 5–10 years old were considered middle‐aged, and shrubs more than 10 years old were considered mature plantings. For the rodent trapping method, the number of caught rodents < 9 was considered mild, values from 10 to 19 were considered moderate, and > 20 was considered severe. For the survey of damaged plants, a damage rate < 30% was considered mild, a damage rate of 30–49% was considered moderate, and a damage rate > 50% was considered severe. Among these two indicators, the indicator with the highest severity level was chosen to represent the activity of *R. opimus* in that region. Since vegetation and activity are degree indicators, values of 1, 2, and 3 were assigned before model construction to represent the progression of shrubs from young to mature and the increase in activity from mild to severe.

### 2.4 PCA

Due to the large variety of model factors, PCA was needed before model construction to reduce the collinearity of the model, retain relatively more original variables, and eliminate any correlations (Dormann et al. 2013). The resulting principal component factors and their calculations are shown in the Supporting Information.

### 2.5 PSO‐ELM Prediction Model

Since the initial input weights and threshold of the ELM are determined randomly, the training accuracy and time are affected by randomness. Therefore, PSO was used to optimize the initial input weights and threshold of the ELM. All particles in the PSO algorithm have their own position and velocity parameters. The velocity is used to determine the flight distance and direction, and the velocity is dynamically adjusted based on self‐experience and collective experience. In each iteration, the optimal position $p_{\text{best}}$ passed by each particle and the best position $g_{\text{best}}$ found by the swarm is determined. By tracking and updating these two optimal positions, the updated particle velocity and position can be expressed as:

(1) $$v_{i,j}\left(t+1\right)=v_{i,j}\left(t\right)+c_{1}r_{1}\left[p_{i,j}-x_{i,j}\left(t\right)\right]+c_{2}r_{2}\left[p_{g,j}-x_{i,j}\left(t\right)\right]$$

(2) $$x_{i,j}\left(t+1\right)=x_{i,j}\left(t\right)+v_{i,j}\left(t+1\right),j=1,2,\ldots ,D$$

where *c*_1_ and *c*_2_ are learning factors, which represent self‐experience and collective experience, respectively, also known as acceleration constants; *r*_1_ and *r*_2_ are random numbers between 0 and 1; and D is the search dimension, that is, the number of parameters to be optimized.

The PSO‐ELM prediction model needs to initialize the particle swarm; that is, the size and search dimension of the particle swarm should be determined. If the population is too small, it can easily fall into a local optimum, while a large population could affect the optimization time. The input layer weight and hidden layer threshold corresponding to each particle are substituted into the ELM training algorithm. Fitness function of the *i*‐th particle $f_{i}$ is expressed as the reciprocal of the mean square error (MSE). For each particle, its current fitness $f_{i}$ is compared with $f\left(p_{\text{best}}\right)$, and $f_{i}>f\left(p_{\text{best}}\right)$ indicates that the current fitness is higher; then, the current position is used to update the individual historical best position $p_{\text{best}}$; otherwise, $p_{\text{best}}$ remains unchanged. Similarly, after comparing the current fitness $f_{i}$ with $f\left(p_{\text{best}}\right)$, when $f_{i}>f\left(p_{\text{best}}\right)$, the global best position $g_{\text{best}}$ should be updated. Then, the velocity and position of each particle are updated according to Equations (1) and (2). The optimization process is stopped when the number of iterations reaches the maximum number of iterations or the best fitness reaches the set threshold. After the optimal input weight ω and threshold *b* are obtained by the PSO algorithm, they are substituted into formula $\beta =H^{+}T$ using the ELM training algorithm to calculate the model predictions.

### 2.6 Simulation Analysis of the Prediction Results

The BP neural network was selected for comparison with the PSO‐ELM model. In the MATLAB environment, the BP network was created by the newff function, the six components after dimensionality reduction were used as input, the output was the activity level of *R. opimus* , the number of hidden layer nodes was set to 50, the number of hidden layers was 1, the number of training iterations was 1000, the target accuracy was 0.0001, the learning rate was 0.1, and the maximum number of failures was 200. In the samples, 80 sets of data were randomly selected as learning samples, and other data were used as testing samples. The obtained test samples were compared with the real data to determine the prediction accuracy of the two models.

### 2.7 Ethics Statement

The field survey of *R. opimus* was approved by the National Forestry and Grassland Administration (China National Wildlife Management Agency) (KJZXXP202212), and the survey was conducted in accordance with the Technical Regulations of China on Forestry Pest rodents.

## 3 Results

From 2020 to 2022, a total of 138 rodent activity records were collected at 46 sampling sites, resulting in the generation of 92 sets of data on the basis of fall activity records from the previous year. The survey details of all the sampling sites are shown in Table S1.

### 3.1 Influencing Factor Correlation Analysis

**FIGURE 3:**
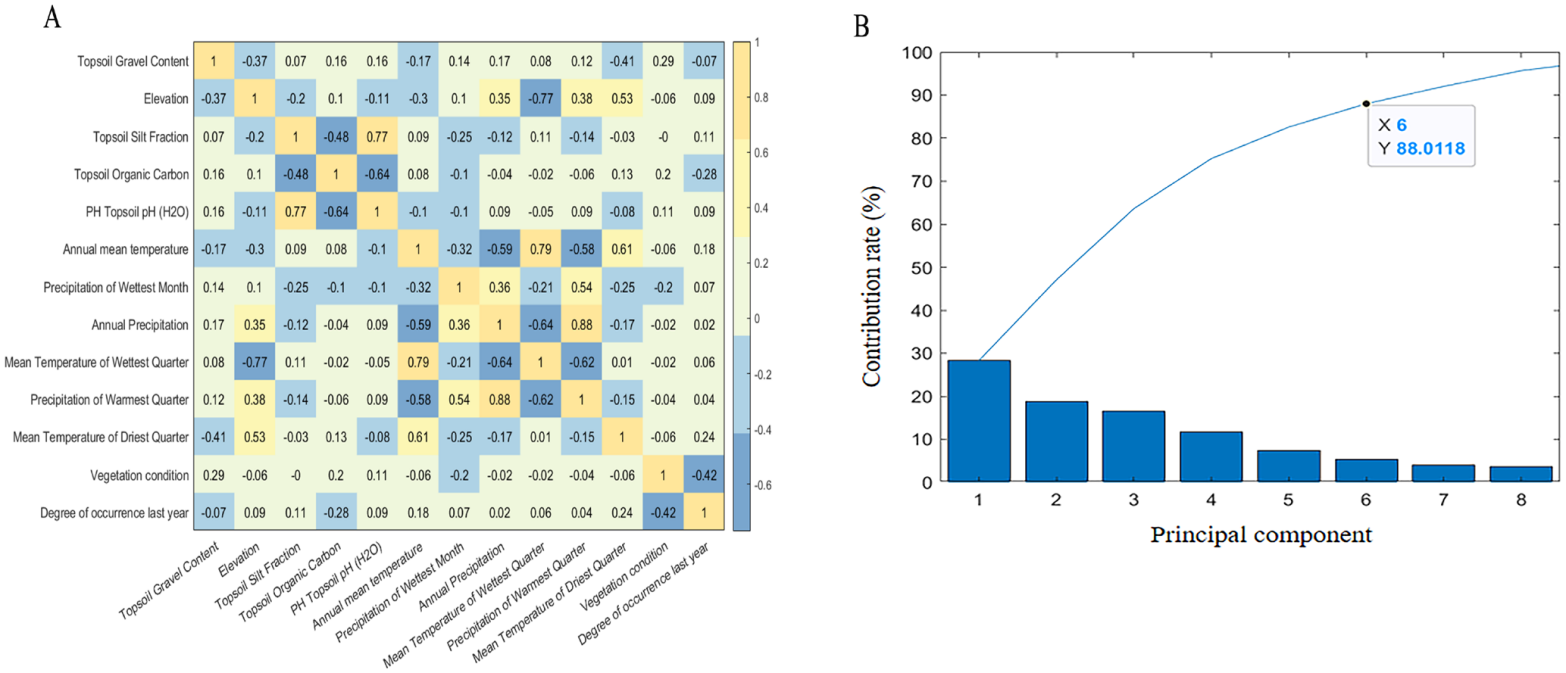
Principal component analysis. (A) is the heatmap of the correlation coefficient, with yellow representing a high correlation and blue representing a low correlation. (B) shows the fitted contribution rate of the new components.

All 13 influencing factors were used as feature inputs, and the Pearson correlation analysis was performed (Figure 3). The correlation coefficients of the samples among the various input features varied significantly, indicating that the contribution rate of each factor to the output was inconsistent. The collinearity among indicators needed to be eliminated, and the dimensionality of the data and the complexity of the prediction model needed to be reduced. The contribution rate of the principal components was set to no less than 85%. All the factors were fitted according to the coefficients. When six new principal components were included, the cumulative contribution rate reached 88.01%, which satisfied the needs of the model. The factor proportions and combinations of the new components are shown in Table S2.

### 3.2 Prediction Model Construction

**FIGURE 4:**
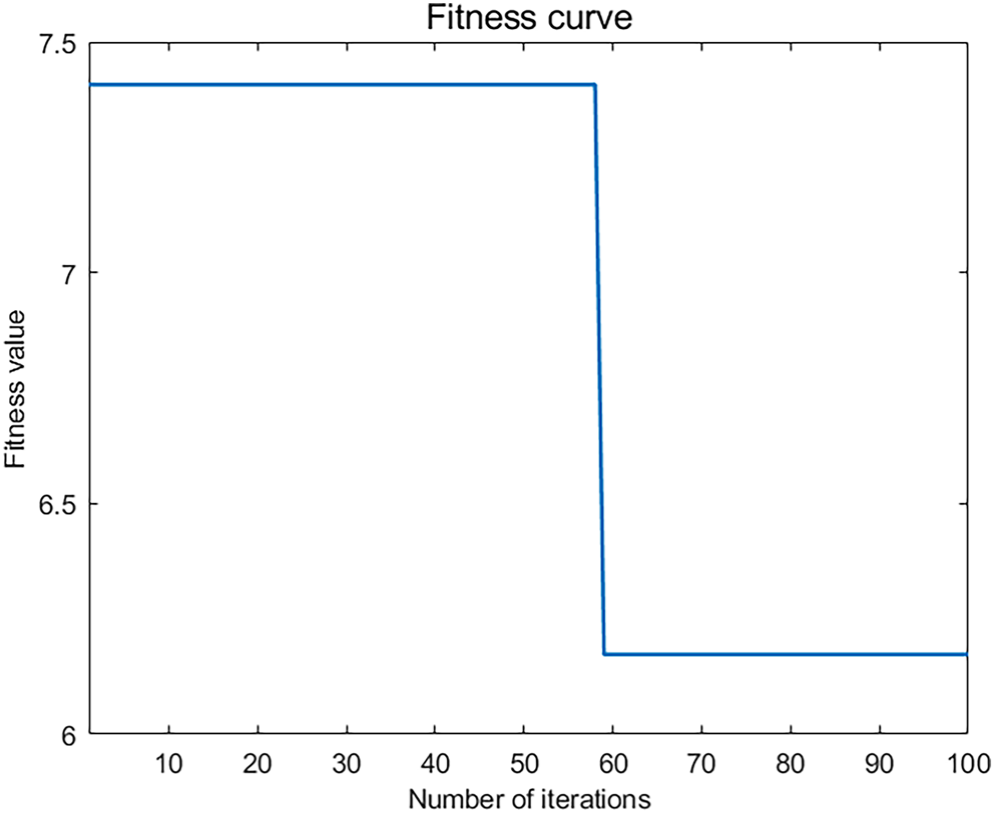
Fitness as a function of the number of iterations of the PSO algorithm.

After dimension reduction, the new components were used to predict the activity level of *R. opimus* on the basis of the sample data. The first 80 samples constituted the training set, and the last 12 samples were used as the test set. The ELM was trained by PSO. The parameters of the PSO particles were as follows: the number of search dimensions, dim, was 2; the population size, pop, was 20; the maximum number of iterations, maximum, was 100; the inertia weight, w, was 0.9; the learning factors c1 = 2 and c2 = 2; the velocity interval was [−10,10]; and the position interval was [−5,5]. The fitness function is shown in Figure 4. In the PSO algorithm, the number of iterations was set to 100. The convergence of the model was very fast, and the algorithm reached convergence after approximately 60 iterations, indicating that the algorithm quickly finds the optimal solution. The ELM was directly used to create a prediction model based on 80 training data points, with the sigmoid function as the activation function. When the number of hidden layer nodes was set to 25, the prediction model achieved good generalization performance.

### 3.3 Simulation Analysis

**FIGURE 5:**
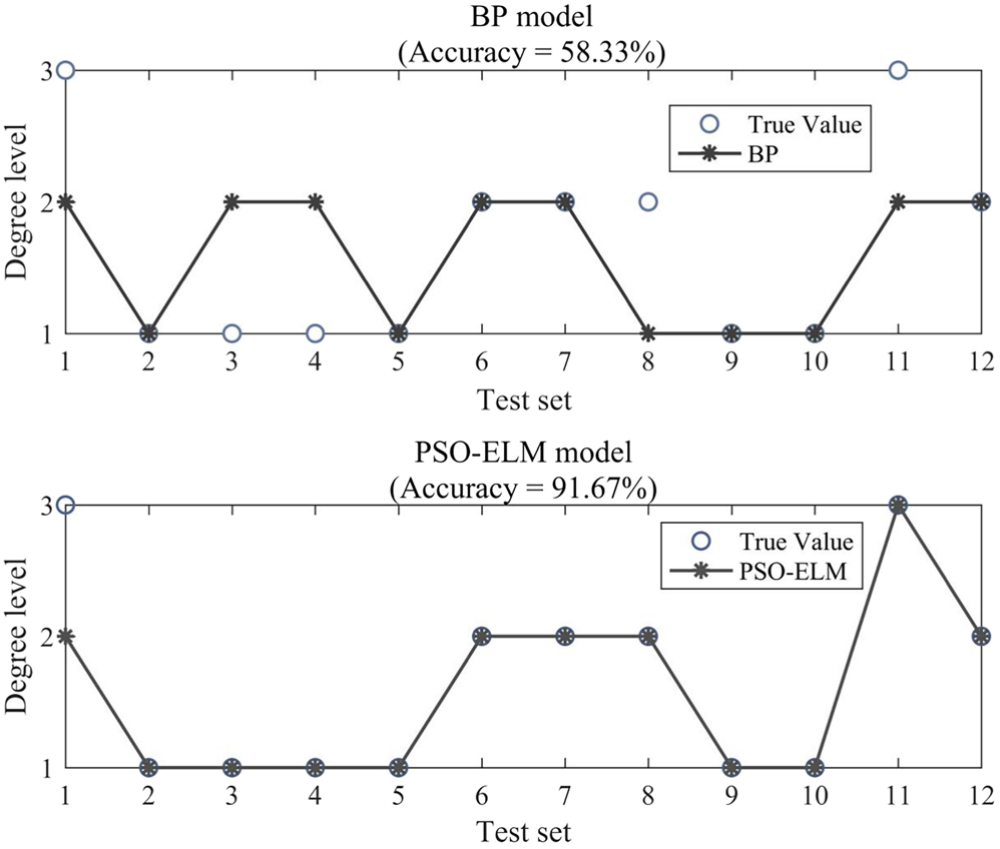
Comparison of the PSO‐ELM and BP neural network simulations. The overlap between the circles and the asterisks in the figure indicates that the prediction is accurate; otherwise, the prediction fails. The degree levels 1, 2, and 3 in the figure indicate that *R. opimus* activity is mild, moderate, and severe, respectively.

In the survey results, 12 sets of data were randomly selected to verify the accuracy of the BP neural network and PSO‐ELM model for predicting the activity level of *R. opimus* . The simulation results of the two models are shown in Figure 5. The prediction accuracy rate of the BP neural network was only 58.33%, and the inaccurate predictions were obtained at the three activity levels of *R. opimus* : high, medium, and low. Compared with that of the BP neural network, the accuracy of the PSO‐ELM model was significantly improved, reaching 91.67%. The increase in accuracy was due to the use of an intelligent optimization algorithm to optimize the parameters as the optimal parameters of the ELM model had already been found. However, the PSO‐ELM still cannot make 100% accurate predictions in cases with high *R. opimus* activity.

## 4 Discussion

For *R. opimus* , the destruction of the environment and vegetation in desert areas is the main reason for its management. However, notably, *R. opimus* is also an important decomposer and primary consumer in the ecosystem and a major food source for predators, such as *Buteo rufinus, Vormela peregusna*, and *Vulpes vulpes* (Wen et al. 2020). On the other hand, when adverse conditions, such as cold and drought conditions, are unfavorable for vegetation growth, *R. opimus* feeding habits could reduce plant transpiration and thus protect plants (Zhang et al. 2009). Therefore, in the management of *R. opimus*, population size needs to be viewed dialectically. Population growth should only be contained when an outbreak is imminent rather than be permanently suppressed through the use of large amounts of chemicals.

At present, the monitoring of *R. opimus* mainly relies on annual surveys in spring and fall. However, the existing spring surveys hardly reflect the number of rodents in fall (Liu et al. 2012). As a result, *R. opimus* may reach large numbers before overwintering; when a larger number of *R. opimus* store food for the coming winter, desert vegetation can lose water and die (Zhao et al. 2021). In this study, a simple prediction method for predicting the activity level of rodents in fall and taking control measures in advance to reduce the risk of disasters is proposed. Through a comparative analysis between the PSO‐ELM and BP neural network prediction results, it was found that the PCA‐PSO‐ELM model has significant advantages. This integrated model requires fewer parameter configurations and involves less computational complexity while enhancing the network's generalizability (Anupam and Pani 2020). Furthermore, it displays an improved convergence speed and higher computational accuracy compared with conventional methods (Hamadani et al. 2023). The favorable adaptability and operational efficiency of the model provide a novel approach for predicting future infestation levels of *R. opimus* . These technical advancements highlight the model's potential as an effective computational tool for ecological risk assessment and pest management applications.

Although our model predicted *R. opimus* activity levels with 92% accuracy, importantly, there are still limitations in its application. One limitation is that the prediction of the high activity level of *R. opimus* by the model was not accurate enough. The main reason for this result is that high‐activity data are scarce, and the samples and training are severely insufficient. Therefore, sample data need to be further enriched to reduce model overfitting due to noisy data (Figueiredo and Ludermir 2014). Another obvious limitation is that only three categories, that is, climate, vegetation, and the base number of rodents, were considered in this study; however, in the model, accurately reflecting the effects of predators on rodent populations through technical means is currently difficult (Randler and Kalb 2020; Cano‐Martíne et al. 2021).

The instability of the rodent population cycle and outbreaks has typically been the focus of population ecology (Jacob et al. 2004; Imholt et al. 2017). In the past, scholars used empirical modeling or ecological models to reflect the dynamic seasonal structure of rodents (Bjørnstad et al. 1995; Shilova and Tchabovsky 2009), resulting in many difficult‐to‐understand biological parameters (Stenseth et al. 2003); this approach greatly constrains the practical application of population dynamics models. In contrast, the neural network method does not need to detail the effect of each biological parameter on population dynamics. In the neural network, all the reproduction factors are used only as neuronal nodes, and the optimal prediction result is obtained through repeated learning and training. From an ecological point of view, neural networks are more inclined to nonlinear equations, and their ability to explain the driving forces of population dynamics is poor. However, as far as application is concerned, the neural networks with excellent multi‐interference processing and convenient operation can still be an important supplement to population dynamics models, and good practical results have been achieved on single‐solution problems (Zhang et al. 2024).

In our study, artificial intelligence technology, particularly neural networks, is demonstrated to have significant potential for pest management. This technology not only reduces the time required for field investigations and minimizes researchers' exposure to pest rodents but also aids in predicting the risk of rodent infestations, allowing for the implementation of targeted preventive measures in advance. To further increase the prediction accuracy of the model, it is essential to continue collecting activity records for *R. opimus* , especially during periods of high activity, in future studies. This will improve the model's training ability and ability to predict high outbreak risk. Additionally, it is important to consider other biological factors, such as predator interactions and the activities of other rodent species, that may influence *R. opimus* populations, and these variables should be incorporated into the modeling process. Furthermore, the findings of this study can be applied to enhance management in other areas where *R. opimus* occurs, and validation tests must be conducted to improve the model's accuracy.

## 5 Conclusions

In summary, based on a three‐year survey at 46 *R. opimus* monitoring sites in China, a PSO‐ELM prediction model suitable for determining the severity of *R. opimus* infestations was developed. Notably, the prediction accuracy of the proposed model was greater than that of the BP neural network model. The proposed method can be used to determine the activity level of *R. opimus* in the event of a potential large‐scale infestation and provides a reference for management departments to develop rodent control strategies.

## Author Contributions

**Fan Jiang:** conceptualization (equal), data curation (equal), formal analysis (equal), methodology (equal), software (equal), validation (equal), visualization (equal), writing – original draft (equal). **Peng Peng:** conceptualization (equal), formal analysis (equal), writing – original draft (equal). **Zhenting Xu:** methodology (equal), validation (equal). **Yu Xu:** methodology (equal), validation (equal). **Ding Yang:** formal analysis (equal), writing – original draft (equal). **Shouquan Chai:** formal analysis (equal), writing – original draft (equal). **Shuai Yuan:** formal analysis (equal), writing – original draft (equal). **Limin Hua:** conceptualization (equal), supervision (equal), writing – original draft (equal). **Dawei Wang:** formal analysis (equal), software (equal), writing – original draft (equal). **Xuanye Wen:** conceptualization (equal), funding acquisition (equal), supervision (equal), writing – review and editing (equal).

## Conflicts of Interest

The authors declare no conflicts of interest.

## Supporting information


Appendix S1.

## Data Availability

The data that support the findings of this study are openly available in Dryad. https://doi.org/10.5061/dryad.0p2ngf29t.
