# Supplementary material for: Predicting the Activity Level of the Great Gerbil ( Rhombomys opimus ) via Machine Learning
Source: Ecol Evol. 2025 May 26;15(5):e71452. doi: 10.1002/ece3.71452 (PMC12104662; doi:10.1002/ece3.71452)
Supplement: Supplementary file 1 — Appendix S1. [file ECE3-15-e71452-s001.doc]

Supplementary materials for

**Predicting the hazard level of the great gerbil (*Rhombomys opimus*) via machine learning**

Contents:

1. Steps for Calculating Principal Component Analysis (PCA).
2. Talbe S1. The vegetation, environment and occurrence of *Rhombomys opimus* in the survey site.
3. Table S2. Principal component coefficient.

**Steps for Calculating PCA.**

In this study, the following six steps were adopted:

1) The original data matrix $X_{n\times p}$is constructed for the variable sampling.

$X_{n\times p}=\left[\begin{matrix} x_{11} & x_{12} & \cdots& x_{1p} \\ x_{21} & x_{22} & \cdots& x_{2p} \\ \cdots& \cdots& \cdots& \cdots\\ x_{n1} & x_{n2} & \cdots& x_{np} \end{matrix}\right]$ (1)

where *n* is the number of samples and *p* is the number of indicators in samples.

2) Normalization is performed on $X_{n\times p}$to obtain the standardized matrix $X_{n\times p}^{*}$.

3) For the standardized matrix$X_{n\times p}^{*}$, the correlation coefficient matrix ***R*** is solved.

The calculation formula of the correlation coefficient is as follows:

(2)

where $r_{ij}$ indicates that a correlation coefficient is between $x_{i}$ and $x_{j}$, and $r_{ij}=r_{ji}$.

4) The eigenequation of the sample correlation coefficient matrix ***R*** is solved to obtain the eigenvalues and eigenvectors***.*** To solve the eigenvalues and eigenvectors, first the eigenequation $\left|\lambda E-R\right|=0\text{，}\lambda_{i}(i=1\text{，}2\text{，}\cdots\text{，}p)$is solved, and the *p* eigenvalues are arranged in descending order, that is, $\lambda_{1}\geq\lambda_{2}\geq\lambda_{3}\cdots\geq\lambda_{p}\geq0$; second, the$(\lambda_{i}E-R)X=0$is solved to find the *X* corresponding to each $\lambda_{i}$.

5) The cumulative contribution rate is obtained, and the number of principal components is preliminarily estimated. The number of selected principal components should make the cumulative contribution rate reach 85%. The number of selected principal components depends on the cumulative contribution rate of the principal components, and the contribution rate $Q_{i}$ and the cumulative contribution rate $Q_{_{\sum}}\text{（}m\text{）}$of a principal component are calculated as follows:

$Q_{i}=\frac{\lambda_{i}}{\sum_{k=1}^{p} \lambda_{k}}\times100\%\text{ }(i=1,2,\cdots,p)$ (3)

$Q_{_{\sum}}\text{（}m\text{）}=\sum_{k=1}^{m} Q_{k}\text{ }(m=1,2,\cdots,p)$ (4)

6) The principal component loadings and principal component scores are calculated. The principal component loading is the loading factor corresponding to each principal component, and the calculation formula for the principal component loading is:

$l_{ij}=\sqrt{\lambda_{i}}e_{ij}(i,j=1,2,\cdots,p)$ (5)

The principal component score can be calculated by substituting the original sample data or standardized sample data into the Equation 5 for principal components, and the calculation formula is:

(6)

**Talbe S1. The vegetation, environment and occurrence of *Rhombomys opimus* in the survey site.**

| Number | Topsoil Gravel Content  (%) | Elevation  (m) | Topsoil Silt Fraction  (%) | Topsoil Organic Carbon  (%) | PH Topsoil pH (H2O)  -log(H+) | Annual mean temperature  (℃) | Precipitation of Wettest Month (july)  (mm) | Annual Precipitation  (mm) | Mean Temperature of Wettest Quarter  (℃) | Precipitation of Warmest Quarter  (℃) | Mean Temperature of Driest Quarter  (℃) | Vegetation condition | Degree of occurrence last year | Autumn actual degree |
| --- | --- | --- | --- | --- | --- | --- | --- | --- | --- | --- | --- | --- | --- | --- |
|
| 1 | 10 | 1227 | 10 | 0.43 | 6.7 | 10.4 | 10.7 | 114.7 | 25.2 | 34.2 | -6.4 | 2 | 3 | 3 |
| 2 | 7 | 1022 | 44 | 0.49 | 7.7 | 10.5 | 29.5 | 215.8 | 24.2 | 114.3 | -5.6 | 1 | 3 | 2 |
| 3 | 10 | 1366 | 29 | 0.58 | 8.1 | 7.2 | 49.1 | 205.2 | 21.8 | 111.6 | -9.9 | 1 | 2 | 3 |
| 4 | 7 | 1135 | 44 | 0.49 | 7.7 | 9.7 | 5.2 | 78.9 | 23.7 | 38.1 | -6.4 | 2 | 3 | 2 |
| 5 | 10 | 1037 | 50 | 1.12 | 7.8 | 9.5 | 6.0 | 79.2 | 22.9 | 16.4 | -6.3 | 1 | 3 | 2 |
| 6 | 10 | 1573 | 6 | 0.43 | 5.7 | 10.3 | 54.3 | 227.3 | 23.8 | 96.1 | -4.9 | 1 | 2 | 2 |
| 7 | 2 | 1538 | 29 | 0.47 | 7.0 | 10.5 | 0.0 | 115.6 | 24.2 | 31.4 | -4.9 | 1 | 3 | 3 |
| 8 | 15 | 936 | 48 | 0.60 | 8.0 | 11.1 | 1.7 | 11.5 | 26.4 | 2.2 | -6.6 | 2 | 2 | 3 |
| 9 | 10 | 2016 | 50 | 1.12 | 7.8 | 6.4 | 6.3 | 361.9 | 18.5 | 127.2 | -7.3 | 1 | 2 | 3 |
| 10 | 10 | 1434 | 6 | 2.41 | 5.9 | 10.5 | 13.7 | 212.1 | 23.1 | 67.3 | -4.0 | 2 | 1 | 1 |
| 11 | 10 | 1630 | 42 | 1.15 | 8.2 | 10.5 | 5.5 | 206.9 | 22.7 | 90.9 | -3.3 | 3 | 1 | 1 |
| 12 | 10 | 1523 | 50 | 1.12 | 7.8 | 8.6 | 3.9 | 191.9 | 21.7 | 57.8 | -6.8 | 3 | 1 | 1 |
| 13 | 10 | 1367 | 50 | 1.12 | 7.8 | 10.9 | 3.9 | 100.7 | 24.1 | 50.6 | -4.4 | 2 | 2 | 2 |
| 14 | 7 | 2106 | 38 | 0.46 | 8.1 | 7.4 | 15.8 | 405.1 | 19.1 | 142.4 | -5.8 | 1 | 2 | 1 |
| 15 | 4 | 1475 | 46 | 0.42 | 7.9 | 9.0 | 5.3 | 118.3 | 22.6 | 47.4 | -6.8 | 1 | 1 | 1 |
| 16 | 7 | 2503 | 38 | 0.46 | 8.1 | 4.8 | 31.0 | 217.8 | 16.6 | 101.1 | -8.5 | 2 | 1 | 1 |
| 17 | 10 | 2290 | 6 | 2.41 | 5.9 | 5.3 | 16.9 | 312.9 | 17.2 | 119.8 | -8.4 | 3 | 2 | 1 |
| 18 | 10 | 1449 | 6 | 2.41 | 5.9 | 9.7 | 2.6 | 98.9 | 23.1 | 37.0 | -5.6 | 2 | 1 | 1 |
| 19 | 10 | 1347 | 50 | 1.12 | 7.8 | 9.5 | 3.6 | 98.3 | 23.2 | 35.4 | -6.5 | 1 | 1 | 1 |
| 20 | 10 | 1782 | 6 | 2.41 | 5.9 | 8.3 | 3.1 | 184.8 | 20.7 | 53.4 | -6.2 | 3 | 1 | 1 |
| 21 | 10 | 1453 | 50 | 1.12 | 7.8 | 9.0 | 25.0 | 112.8 | 22.2 | 37.7 | -6.1 | 3 | 1 | 1 |
| 22 | 10 | 1272 | 50 | 1.12 | 7.8 | 10.0 | 13.3 | 64.3 | 23.9 | 21.0 | -6.0 | 2 | 3 | 2 |
| 23 | 2 | 2178 | 29 | 0.47 | 7.0 | 7.0 | 15.5 | 138.5 | 18.6 | 44.5 | -5.8 | 2 | 1 | 1 |
| 24 | 2 | 1670 | 29 | 0.47 | 7.0 | 10.9 | 1.0 | 49.3 | 25.3 | 2.2 | -4.8 | 1 | 3 | 3 |
| 25 | 10 | 1475 | 6 | 2.41 | 5.9 | 8.5 | 5.7 | 50.4 | 22.2 | 13.4 | -6.8 | 3 | 1 | 1 |
| 26 | 10 | 1111 | 50 | 1.12 | 7.8 | 11.4 | 1.0 | 146.4 | 24.6 | 20.9 | -3.3 | 2 | 2 | 1 |
| 27 | 10 | 384 | 50 | 1.12 | 7.8 | 12.9 | 0.8 | 24.2 | 28.7 | 10.0 | -5.2 | 3 | 1 | 1 |
| 28 | 7 | 278 | 36 | 0.99 | 7.8 | 15.3 | 0.7 | 5.5 | 32.2 | 4.6 | -4.6 | 3 | 1 | 1 |
| 29 | 10 | 753 | 6 | 2.41 | 5.9 | 11.3 | 6.6 | 51.9 | 27.3 | 23.1 | -7.2 | 2 | 1 | 1 |
| 30 | 10 | 1843 | 29 | 0.58 | 8.1 | 5.6 | 14.9 | 130.1 | 19.4 | 67.4 | -10.3 | 3 | 1 | 1 |
| 31 | 15 | 579 | 48 | 0.60 | 8.0 | 7.9 | 25.2 | 185.0 | 25.5 | 68.6 | -14.6 | 3 | 1 | 1 |
| 32 | 10 | 524 | 6 | 2.41 | 5.9 | 8.6 | 44.1 | 189.5 | 26.0 | 78.8 | -14.0 | 1 | 2 | 1 |
| 33 | 15 | 454 | 48 | 0.60 | 8.0 | 8.3 | 19.9 | 156.6 | 25.1 | 44.1 | -13.7 | 2 | 3 | 2 |
| 34 | 3 | 768 | 42 | 1.26 | 5.7 | 6.7 | 7.9 | 191.5 | 22.9 | 54.5 | -13.7 | 2 | 1 | 1 |
| 35 | 10 | 700 | 50 | 1.12 | 7.8 | 8.4 | 9.7 | 153.1 | 24.7 | 51.4 | -12.4 | 1 | 1 | 1 |
| 36 | 10 | 1221 | 15 | 0.41 | 8.3 | 6.7 | 10.5 | 343.6 | 21.2 | 70.6 | -9.7 | 3 | 1 | 1 |
| 37 | 10 | 332 | 6 | 0.43 | 5.7 | 9.1 | 11.3 | 69.6 | 26.9 | 34.6 | -14.3 | 1 | 1 | 1 |
| 38 | 10 | 316 | 50 | 1.12 | 7.8 | 9.0 | 6.8 | 76.2 | 26.1 | 30.3 | -14.4 | 3 | 1 | 1 |
| 39 | 4 | 967 | 46 | 0.42 | 7.9 | 11.7 | 7.5 | 41.4 | 25.7 | 14.2 | -4.4 | 3 | 1 | 1 |
| 40 | 2 | 1105 | 44 | 0.41 | 7.9 | 10.6 | 6.8 | 40.3 | 25.4 | 16.3 | -6.3 | 1 | 2 | 2 |
| 41 | 15 | 496 | 48 | 0.60 | 8.0 | 8.1 | 15.5 | 155.4 | 24.9 | 35.4 | -14.1 | 2 | 1 | 1 |
| 42 | 15 | 521 | 48 | 0.60 | 8.0 | 8.4 | 4.6 | 339.7 | 23.1 | 80.4 | -10.1 | 2 | 3 | 2 |
| 43 | 10 | 1079 | 29 | 0.58 | 8.1 | 7.2 | 19.2 | 281.5 | 21.0 | 146.9 | -8.9 | 3 | 2 | 2 |
| 44 | 10 | 477 | 29 | 0.58 | 8.1 | 5.7 | 9.9 | 137.4 | 21.7 | 39.1 | -14.6 | 1 | 1 | 1 |
| 45 | 10 | 815 | 29 | 0.58 | 8.1 | 5.4 | 17.5 | 264.4 | 22.2 | 103.7 | -15.5 | 3 | 1 | 1 |
| 46 | 15 | 499 | 48 | 0.60 | 8.0 | 5.5 | 10.1 | 119.8 | 23.0 | 52.8 | -16.5 | 3 | 1 | 1 |
| 47 | 10 | 1227 | 10 | 0.43 | 6.7 | 10.4 | 10.7 | 114.7 | 25.2 | 34.2 | -6.4 | 2 | 3 | 2 |
| 48 | 7 | 1022 | 44 | 0.49 | 7.7 | 10.5 | 29.5 | 215.8 | 24.2 | 114.3 | -5.6 | 1 | 2 | 2 |
| 49 | 10 | 1366 | 29 | 0.58 | 8.1 | 7.2 | 49.1 | 205.2 | 21.8 | 111.6 | -9.9 | 1 | 3 | 3 |
| 50 | 7 | 1135 | 44 | 0.49 | 7.7 | 9.7 | 5.2 | 78.9 | 23.7 | 38.1 | -6.4 | 2 | 2 | 1 |
| 51 | 10 | 1037 | 50 | 1.12 | 7.8 | 9.5 | 6.0 | 79.2 | 22.9 | 16.4 | -6.3 | 1 | 2 | 2 |
| 52 | 10 | 1573 | 6 | 0.43 | 5.7 | 10.3 | 54.3 | 227.3 | 23.8 | 96.1 | -4.9 | 1 | 2 | 2 |
| 53 | 2 | 1538 | 29 | 0.47 | 7.0 | 10.5 | 0.0 | 115.6 | 24.2 | 31.4 | -4.9 | 1 | 3 | 3 |
| 54 | 15 | 936 | 48 | 0.60 | 8.0 | 11.1 | 1.7 | 11.5 | 26.4 | 2.2 | -6.6 | 2 | 3 | 2 |
| 55 | 10 | 2016 | 50 | 1.12 | 7.8 | 6.4 | 6.3 | 361.9 | 18.5 | 127.2 | -7.3 | 1 | 3 | 3 |
| 56 | 10 | 1434 | 6 | 2.41 | 5.9 | 10.5 | 13.7 | 212.1 | 23.1 | 67.3 | -4.0 | 2 | 1 | 1 |
| 57 | 10 | 1630 | 42 | 1.15 | 8.2 | 10.5 | 5.5 | 206.9 | 22.7 | 90.9 | -3.3 | 3 | 1 | 1 |
| 58 | 10 | 1523 | 50 | 1.12 | 7.8 | 8.6 | 3.9 | 191.9 | 21.7 | 57.8 | -6.8 | 3 | 1 | 2 |
| 59 | 10 | 1367 | 50 | 1.12 | 7.8 | 10.9 | 3.9 | 100.7 | 24.1 | 50.6 | -4.4 | 2 | 2 | 1 |
| 60 | 7 | 2106 | 38 | 0.46 | 8.1 | 7.4 | 15.8 | 405.1 | 19.1 | 142.4 | -5.8 | 1 | 1 | 1 |
| 61 | 4 | 1475 | 46 | 0.42 | 7.9 | 9.0 | 5.3 | 118.3 | 22.6 | 47.4 | -6.8 | 1 | 1 | 1 |
| 62 | 7 | 2503 | 38 | 0.46 | 8.1 | 4.8 | 31.0 | 217.8 | 16.6 | 101.1 | -8.5 | 2 | 1 | 1 |
| 63 | 10 | 2290 | 6 | 2.41 | 5.9 | 5.3 | 16.9 | 312.9 | 17.2 | 119.8 | -8.4 | 3 | 1 | 1 |
| 64 | 10 | 1449 | 6 | 2.41 | 5.9 | 9.7 | 2.6 | 98.9 | 23.1 | 37.0 | -5.6 | 2 | 1 | 1 |
| 65 | 10 | 1347 | 50 | 1.12 | 7.8 | 9.5 | 3.6 | 98.3 | 23.2 | 35.4 | -6.5 | 1 | 1 | 2 |
| 66 | 10 | 1782 | 6 | 2.41 | 5.9 | 8.3 | 3.1 | 184.8 | 20.7 | 53.4 | -6.2 | 3 | 1 | 1 |
| 67 | 10 | 1453 | 50 | 1.12 | 7.8 | 9.0 | 25.0 | 112.8 | 22.2 | 37.7 | -6.1 | 3 | 1 | 1 |
| 68 | 10 | 1272 | 50 | 1.12 | 7.8 | 10.0 | 13.3 | 64.3 | 23.9 | 21.0 | -6.0 | 2 | 2 | 1 |
| 69 | 2 | 2178 | 29 | 0.47 | 7.0 | 7.0 | 15.5 | 138.5 | 18.6 | 44.5 | -5.8 | 2 | 1 | 1 |
| 70 | 2 | 1670 | 29 | 0.47 | 7.0 | 10.9 | 1.0 | 49.3 | 25.3 | 2.2 | -4.8 | 1 | 3 | 2 |
| 71 | 10 | 1475 | 6 | 2.41 | 5.9 | 8.5 | 5.7 | 50.4 | 22.2 | 13.4 | -6.8 | 3 | 1 | 1 |
| 72 | 10 | 1111 | 50 | 1.12 | 7.8 | 11.4 | 1.0 | 146.4 | 24.6 | 20.9 | -3.3 | 2 | 1 | 1 |
| 73 | 10 | 384 | 50 | 1.12 | 7.8 | 12.9 | 0.8 | 24.2 | 28.7 | 10.0 | -5.2 | 3 | 1 | 1 |
| 74 | 7 | 278 | 36 | 0.99 | 7.8 | 15.3 | 0.7 | 5.5 | 32.2 | 4.6 | -4.6 | 3 | 1 | 1 |
| 75 | 10 | 753 | 6 | 2.41 | 5.9 | 11.3 | 6.6 | 51.9 | 27.3 | 23.1 | -7.2 | 2 | 1 | 1 |
| 76 | 10 | 1843 | 29 | 0.58 | 8.1 | 5.6 | 14.9 | 130.1 | 19.4 | 67.4 | -10.3 | 3 | 1 | 1 |
| 77 | 15 | 579 | 48 | 0.60 | 8.0 | 7.9 | 25.2 | 185.0 | 25.5 | 68.6 | -14.6 | 3 | 1 | 1 |
| 78 | 10 | 524 | 6 | 2.41 | 5.9 | 8.6 | 44.1 | 189.5 | 26.0 | 78.8 | -14.0 | 1 | 1 | 1 |
| 79 | 15 | 454 | 48 | 0.60 | 8.0 | 8.3 | 19.9 | 156.6 | 25.1 | 44.1 | -13.7 | 2 | 2 | 2 |
| 80 | 3 | 768 | 42 | 1.26 | 5.7 | 6.7 | 7.9 | 191.5 | 22.9 | 54.5 | -13.7 | 2 | 1 | 1 |
| 81 | 10 | 700 | 50 | 1.12 | 7.8 | 8.4 | 9.7 | 153.1 | 24.7 | 51.4 | -12.4 | 1 | 1 | 1 |
| 82 | 10 | 1221 | 15 | 0.41 | 8.3 | 6.7 | 10.5 | 343.6 | 21.2 | 70.6 | -9.7 | 3 | 1 | 1 |
| 83 | 10 | 332 | 6 | 0.43 | 5.7 | 9.1 | 11.3 | 69.6 | 26.9 | 34.6 | -14.3 | 1 | 1 | 1 |
| 84 | 10 | 316 | 50 | 1.12 | 7.8 | 9.0 | 6.8 | 76.2 | 26.1 | 30.3 | -14.4 | 3 | 1 | 1 |
| 85 | 4 | 967 | 46 | 0.42 | 7.9 | 11.7 | 7.5 | 41.4 | 25.7 | 14.2 | -4.4 | 3 | 1 | 1 |
| 86 | 2 | 1105 | 44 | 0.41 | 7.9 | 10.6 | 6.8 | 40.3 | 25.4 | 16.3 | -6.3 | 1 | 2 | 2 |
| 87 | 15 | 496 | 48 | 0.60 | 8.0 | 8.1 | 15.5 | 155.4 | 24.9 | 35.4 | -14.1 | 2 | 1 | 1 |
| 88 | 15 | 521 | 48 | 0.60 | 8.0 | 8.4 | 4.6 | 339.7 | 23.1 | 80.4 | -10.1 | 2 | 2 | 1 |
| 89 | 10 | 1079 | 29 | 0.58 | 8.1 | 7.2 | 19.2 | 281.5 | 21.0 | 146.9 | -8.9 | 3 | 2 | 3 |
| 90 | 10 | 477 | 29 | 0.58 | 8.1 | 5.7 | 9.9 | 137.4 | 21.7 | 39.1 | -14.6 | 1 | 1 | 1 |
| 91 | 10 | 815 | 29 | 0.58 | 8.1 | 5.4 | 17.5 | 264.4 | 22.2 | 103.7 | -15.5 | 3 | 1 | 1 |
| 92 | 15 | 499 | 48 | 0.60 | 8.0 | 5.5 | 10.1 | 119.8 | 23.0 | 52.8 | -16.5 | 3 | 1 | 1 |

In the table Vegetation condition values 1,2,3 indicate shrubs age young, middle-aged and mature plantings. Degree of occurrence last year and Autumn actual degree values 1,2,3 indicate *Rhombomys opimus* activity level light to medium-heavy.

**Table S2. Principal component coefficient.**

| Index | y1 | y2 | y3 | y4 | y5 | y6 |
| --- | --- | --- | --- | --- | --- | --- |
| X1 | -0.0548 | 0.2475 | -0.4078 | 0.0491 | -0.5636 | 0.2683 |
| X2 | -0.2864 | -0.3039 | 0.3543 | -0.2788 | -0.014 | 0.011 |
| X3 | 0.1179 | 0.4788 | 0.2339 | -0.2223 | -0.042 | 0.0682 |
| X4 | 0.0091 | -0.4338 | -0.3354 | -0.1211 | -0.238 | 0.2584 |
| X5 | -0.0069 | 0.5344 | 0.2245 | -0.2419 | -0.111 | -0.1129 |
| X6 | 0.4356 | -0.1399 | 0.1456 | 0.0744 | -0.3453 | -0.29 |
| X7 | -0.2771 | 0.0178 | -0.0638 | 0.4623 | -0.074 | -0.5813 |
| X8 | -0.4479 | 0.0558 | 0.0193 | 0.0182 | -0.2478 | 0.0121 |
| X9 | 0.451 | 0.0719 | -0.139 | 0.2636 | -0.1244 | -0.2265 |
| X10 | -0.4598 | 0.0463 | 0.0389 | 0.0896 | -0.2413 | -0.2122 |
| X11 | 0.1338 | -0.3299 | 0.4298 | -0.2209 | -0.3833 | -0.2008 |
| X12 | 0.0119 | 0.047 | -0.323 | -0.5425 | -0.2579 | -0.2862 |
| X13 | 0.0271 | 0.0426 | 0.3933 | 0.3959 | -0.3712 | 0.4502 |

According to the coefficient of each index, the expression of six principal components is obtained, as shown in formula 1-6.

y_1_=-0.0548X_1_-0.2864X_2_+0.1179X_3_+0.0091X_4_-0.0069X_5_+0.4356X_6_-0.2771X_7_-0.4479X_8_+0.4510X_9_-0.4598X_10_+0.1338X_11_+0.0119X_12_+0.0271X_13_; (1)

y_2_=0.2475X_1_-0.3039X_2_+0.4788X_3_-0.4338X_4_+0.5344X_5_+0.1399X_6_+0.0178X_7_+0.0558X_8_+0.0719X_9_+0.0463X_10_-0.3299X_11_+0.0470X_12_+0.0426X_13_ ; (2)

y_3_=-0.4078X_1_+0.3543X_2_+0.2339X_3_-0.3354X_4_+0.2245X_5_+0.1456X_6_-0.0638X_7_+0.0193X_8_-0.1390X_9_+0.0389X_10_+0.4298X_11_-0.3230X_12_+0.3933X_13_ ; (3)

y_4_=0.0491X_1_-0.2788X_2_+-0.2223X_3_-0.1211X_4_-0.2419X_5_+0.0744X_6_+0.4623X_7_+0.0182X_8_+0.2636X_9_+0.0896X_10_-0.2209X_11_-0.5425X_12_-0.3712X_13_ ; (4)

y_5_=-0.5636X_1_-0.0140X_2_-0.0420X_3_-0.2380X_4_-0.1110X_5_-0.3453X_6_-0.0740X_7_-0.2478X_8_-0.1244X_9_-0.2413X_10_-0.3833X_11_-0.2579X_12_+0.3933X_13_ ; (5)

y_6_=0.2683X_1_+0.0110X_2_+0.0682X_3_+0.2584X_4_-0.1129X_5_-0.2900X_6_-0.5813X_7_+0.0121X_8_-0.2265X_9_+-0.2122X_10_-0.2008X_11_-0.2862X_12_+0.4502X_13_ ; (6)
